# A Randomized Clinical Trial of a Functional Electrical Stimulation Mimic to Gait Promotes Motor Recovery and Brain Remodeling in Acute Stroke

**DOI:** 10.1155/2018/8923520

**Published:** 2018-12-18

**Authors:** Xiuyuan Zheng, Danfeng Chen, Tiebin Yan, Dongmei Jin, Zhiqiang Zhuang, Zhimei Tan, Wei Wu

**Affiliations:** ^1^Department of Rehabilitation Medicine, Sun Yat-sen Memorial Hospital, Sun Yat-sen University, Guangzhou, China; ^2^Department of Neurology, Jiangmen Central Hospital, Jiangmen, China; ^3^Guangdong Engineering Technology Research Center for Rehabilitation and Elderly Care, Guangzhou, China; ^4^Department of Rehabilitation Medicine, The First Affiliated Hospital of Zhengzhou University, Zhengzhou, China

## Abstract

Functional electrical stimulation can improve motor function after stroke. The mechanism may involve activity-dependent plasticity and brain remodeling. The aim of our study was to investigate the effectiveness of a patterned electrical stimulation FES mimic to gait in motor recovery among stroke survivors and to investigate possible mechanisms through brain fMRI. Forty-eight subjects were recruited and randomly assigned to a four-channel FES group (*n* = 18), a placebo group (*n* = 15), or a dual-channel FES group (*n* = 15). Stimulation lasted for 30 minutes in each session for 3 weeks. All of the subjects were assessed at baseline and after weeks 1, 2, and 3. The assessments included the Fugl-Meyer Assessment, the Postural Assessment Scale for Stroke Patients, Brunel's Balance Assessment, the Berg Balance Scale, and the modified Barthel Index. Brain fMRI were acquired before and after the intervention. All of the motor assessment scores significantly increased week by week in all the three groups. The four-channel group showed significantly better improvement than the dual-channel group and placebo groups. fMRI showed that fractional anisotropy was significantly increased in both the four-channel and dual-channel groups compared with the placebo group and fiber bundles had increased significantly on the ipsilateral side, but not on the contralateral side in the group given four-channel stimulation. In conclusion, when four-channel FES induces cycling movement of the lower extremities based on a gait pattern, it may be more effective in promoting motor recovery and induce more plastic changes and brain remodeling than two-channel stimulation. This trial is registered with clinical trial registration unique identifier ChiCTR-TRC-11001615.

## 1. Introduction

Hemiparesis is one of the most serious disabling sequelae of stroke. It is ranked as the leading cause of disability and greatly affects the daily activities of stroke survivors [1]. Restoring normal gait after stroke is a key issue for all patients as soon as their medical condition has stabilized. For many years, functional electrical stimulation has been applied as a neuroprosthetic, in which the application of patterned peripheral neuromuscular stimulation evokes functional movements in the absence of voluntary muscle activation [2–4]. FES has also been applied to increase the voluntary drive of motor function [5, 6]. An array of stimulating electrodes with multiple output channels can be individually activated to apply complex stimulation patterns to muscles [7, 8]. Dual-channel and four-channel FES are now commonly employed in clinical application. But FES through more channels can induce more complicated movement. Clinical evidence has suggested that multiple-channel FES-mediated training can reduce motor impairment and improve the walking ability of hemiparetic subjects [9, 10]. The benefits include enhanced muscle strength and endurance, increased sense input, decreased spasticity, and improved gesture control [11–13].

Dual-channel FES can be applied to the dorsiflexors and hamstrings to correct foot drop and knee hyperextension during stance [14, 15] and also be delivered to paretic ankle plantar flexors to dorsiflexors to induce greater swing-phase knee flexion, a larger ankle plantarflexion angle at toe-off, and stronger forward propulsion [8]. It has also been applied to the gluteus medius in the stance phase and the tibialis anterior in the swing phase. With hemiparetic stroke survivors, this can improve the spatiotemporal parameters of gait [15, 16]. Four-channel FES was previously applied in lower extremity rehabilitation by our preliminary study. Yan et al. firstly adopted two dual-channel stimulators with a program timer to form one stimulating unit for FES. Surface electrodes were applied on quadriceps, hamstring, tibialis anterior (TA), and medial gastrocnemius (MG) with the subject lying on his/her side and the affected lower extremity being supported by a sling [11]. Xu et al. and Tan et al. made further efforts to explore the clinical effects of four-channel FES by our own design FES machine based on a gait pattern in walking recovery, and they concluded better walking paradigm during stimulation and better assessment scales after 3 weeks of treatment [8, 9].

The risk of falls during gait retraining needs extensive, time-consuming assistance from a physical therapist or assistant, which also brings us the idea of using a sling for lower extremity weight bearing in a side-lying position to mimic the gait pattern during FES treatment. Some results have shown locomotor training on a treadmill to be substantially more effective than conventional clinical physical therapy alone, with significant improvements in daily activities involving stepping and better gait efficiency; the kinematic pattern of which is very similar to that of walking [17]. Researchers paid more and more attention to the entire and patterned locomotor training method. Some studies showed that treadmill training poststroke is generally considered to be more effective than normal physical training with more durable results [18, 19]. These opinions help us further to explore the method for the entire walking ability based on a gait pattern.

Although obvious clinical effects of four-channel FES were gained previously [9, 10], the mechanisms were not well understood. The mechanisms for FES treatment led to the speculation that the effect is achieved through the reintegration of sensorimotor pathways and corticospinal plasticity [20–22]. We tend to adopt neuroimaging to explore proper mechanisms on the basis of our former studies.

Neuroimaging studies found several white matter characteristics to be related to the extent of motor deficit and to recovery after stroke by diffusion tensor imaging (DTI) [23, 24]. Fractional anisotropy (FA) values derived from diffusion tensor imaging (DTI) differ in the intact and damaged or interrupted tracts of white matter. In adults, FA values for the CST correlate strongly with motor function in both the acute and chronic periods of stroke [25, 26]. DTI as a method of fMRI assessment has become a common method of evaluating recovery of extremity function and the mechanism of rehabilitation interventions. Examining FA values of DTI and observing visualized DTT may help to precisely pinpoint the nature of diffusion differences and thus microstructural white matter changes after treatment.

This study explored the possibility of using FES. FES was applied to generate a locomotion-like gait pattern in stroke survivors lying on their side and supported by slings. Both ambulatory and nonambulatory patients were tested. The intuition was that gait-like motion induced by FES could improve walking ability and boost brain plasticity even in a lying position.

## 2. Methods

In this study, we applied the similar design as reported in the literature including the inclusion and exclusion criteria, treatment protocol but the different outcome measurements with the more focusing on the imaging pictures [9].

### 2.1. Participants

Participants were recruited from an inpatient rehabilitation department in Sun Yat-sen Memorial Hospital between August 2012 and September 2014. The inclusion criteria were a diagnosis of first stroke confirmed by a CT or MR scan and resulting in hemiparesis, less than 3 months since the acute event, age between 35 and 80, an ability to perform cycling leg motions, no apparent neuropathy of the peripheral nerve system, no knee or hip joint limitations, and being at Brunnstrom recovery stage I, II, or IV. Exclusion criteria were progressive stroke, stroke due to a tumor or hypoxic encephalopathy, brain stem or cerebellum lesions, an inability to tolerate stimulation, medical comorbidity, or severe cognitive impairment. All of the subjects signed informed consent forms approved by the human subjects Ethics Committee of Sun Yat-sen University (approval number: 20111012). The same recruited protocol was followed if any subject was dropped out after he or she was admitted into the study.

### 2.2. Design

**Figure 1:**
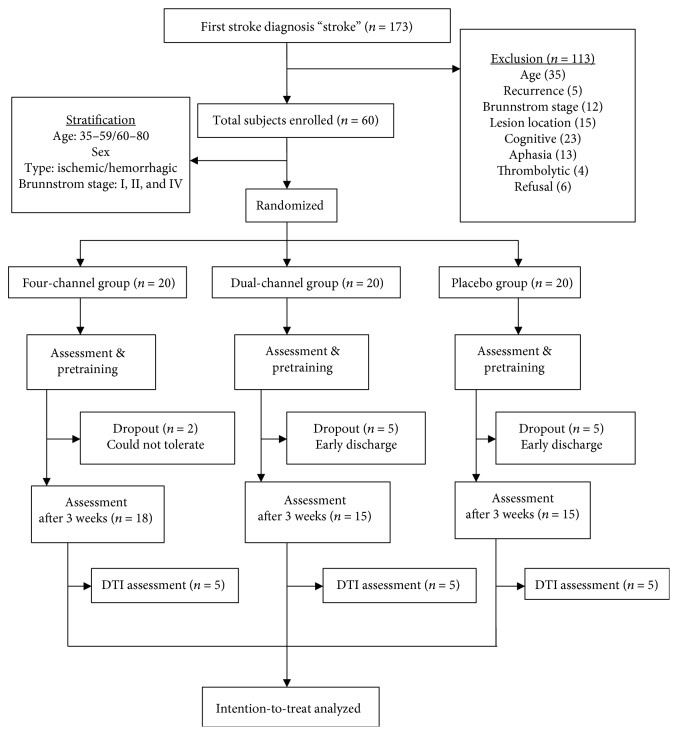
Flowchart of the study.

This was a randomized, placebo-controlled trial. 48 subjects were randomly allocated into a four-channel FES group (FES-4, 18 cases), a dual-channel FES group (FES-2, 15 cases), or a placebo group (FES-P, 15 cases) using a computerized method to minimize the uneven distribution of known variables (Table 1). The stratifications included age (35–59, 60–80) and type of stroke (ischemic, hemorrhagic). Twelve subjects (20%) did not complete the experiments, and another 10 (16.7%) could not undergo physical or imaging assessment. Two subjects (3%) rejected FES treatment in the late phase of the research (Table 1, Figure 1).

### 2.3. Interventions

All of the subjects continued to perform their own rehabilitation standard programs, which involved 2 hours per day of physiotherapy based on neurodevelopmental facilitation and occupational therapy concerning the activities of daily life. The protocols included stretching, muscular conditioning, exercises for trunk control, standing, and walking training. The training was 5 days per week for the 3 weeks of the experiments.

The FES device utilized in the study was designed at Sun Yat-sen Memorial Hospital of Sun Yat-sen University. It has two different systems with two and four channels. In these experiments, the subjects were in a side-lying position during the FES treatment with the hemiplegic leg being supported by two slings. One pattern delivered stimulation through four channels. The two surface electrodes of each channel were placed on the motor points to stimulate the tibialis anterior, quadriceps, hamstrings, and gastrocnemius of the hemiplegic leg in the sequence of the gait cycle. The two-channel group had the tibialis anterior and the peroneus longus and brevis stimulated to facilitate ankle dorsiflexion and eversion in sequence. Those in the FES-P group ostensibly received four-channel FES treatment, but without electricity. A 5-second gait cycle was simulated in the four-channel pattern. The dual-channel pattern was 5 seconds on and 5 seconds off. The intensity of stimulation was increased until a gross muscle contraction was visible and the movements were induced while maintaining the subject's comfort. In both patterns, FES was performed at a frequency of 30 Hz with a pulse width of 0.2 ms.

### 2.4. Outcome Assessment

A therapist blinded to the nature of each subject's treatment assessed lower extremity impairment and activity limitation at baseline and then weekly during the three weeks of intervention.

Lower extremity motor function was assessed with the lower extremity component of the Fugl-Meyer Movement Assessment (FMA) [27] including items dealing with the hip, knee, and ankle. Reflex activity, synergy, movement combining synergies, normal reflexes, and coordination were assessed. The maximum motor performance score was 34 points, with a higher score indicating better performance.

Balance was assessed using the Postural Assessment Scale for Stroke Patients (PASS) [28], Berg's Balance Scale (BBS) [29], and the Brunel Balance Assessment (BBA) [30]. The PASS quantifies the ability to maintain or change a given lying, sitting, or standing posture. It comprises 12 items with 4 levels of increasing difficulty. The maximum score is 36. The BBS consists of 14 five-level items with a total possible score of 56. It quantifies sitting or standing balance. A higher score indicates better postural control. The BBA comprised 12 two-level items with a highest possible score of 12. It quantifies sitting, standing, or walking balance. Three scales were used to alleviate any ceiling and floor effects.

Ability in the activities of daily living was evaluated using the modified Barthel Index (MBI) [31]. It assesses feeding, bathing, grooming, and dressing ability; bowel and bladder control; toilet use; chair-to-bed transfers; mobility; and stair climbing. The total possible score is 100. Subjects with an independent living ability score 60 or more; lower scores represent assisted or totally dependent living.

All of the subjects were scanned at baseline, then weekly, and after the intervention. Functional magnetic resonance images were acquired with an Avanto 1.5T scanner (Siemens, Germany). Twenty consecutive slices (FOV = 230 × 230 mm, thickness/gap = 5 mm/1.5 mm, and matrix = 128 × 128) were obtained using a single-shot, gradient-recalled, echo planar imaging (EPI) sequence (TR = 3200 ms, TE = 105 ms). The diffusion encoding scheme consisted of 30 directions with *b* = 1000 s/mm^2^ and 1 non-diffusion-weighted image (*b* = 0 s/mm^2^). To maintain the consistency of the slices among the scans, the slices of each scan were consistently positioned parallel to a line that joined the most inferior and anterior and inferior and posterior parts of the corpus callosum.

In the examination of each subject, the diffusion tensor images were registered to the corresponding *b* = 0 images with an affine transformation to correct for any eddy current distortion and head motion. The fiber tracts originating from regions of interest (ROIs) were extracted and placed on the ischemic lesion and the homologous area in the unaffected hemisphere at one level of the largest lesion located by the same radiologist. If there were multiple lesions of one cerebral hemisphere, the largest was used in the calculations. The average area of an ROI was 16 mm^2^ or five pixels.

### 2.5. Statistical Analysis

SPSS software (version 18.0) was used in the statistical analysis. Baseline characteristics and scores for the continuous variables were summarized in terms of their means ± standard deviations. Frequencies were used for the categorical variables. Chi-squared tests and analysis of variance (ANOVA) were used as homogeneity tests for the demographic and medical characteristics. Repeated measures analysis of variance was used to test the significance of any effects observed after 1, 2, and 3 weeks of treatment within each group. One-way ANOVA was used to test for between-group comparisons. If there was a significant difference, one-way ANOVA with a post hoc least significant difference test was used to differentiate the significance. Statistical significance was accepted for *p* values less than 0.05.

## 3. Results

Of the sixty-seven patients recruited, forty-eight completed the 3 weeks of treatment and assessment and were included in the analysis. Twelve were dropped out. Table 1 outlines their characteristics and scores before training. There were no significant differences between the groups in terms of the demographic variables or the primary outcome measures (PASS, BBA, BBS, FMA, and MBI) at baseline.

The PASS, BBA, BBS, FMA, and MBI results were all improved significantly week by week in all 3 groups (*p* ≤ 0.05). The MBI scores already showed significantly greater improvement in the four-channel group than in the placebo and dual-channel groups at 1 week of treatment (*p* ≤ 0.001 for both comparisons). But there was no other significant difference between the groups after the first week. The outcomes tended to change after 2 weeks of intervention. Subjects in the four-channel group showed a significant greater improvement in their PASS (*p* ≤ 0.05) and MBI (*p* ≤ 0.05) scores compared to those in the dual FES group. Compared to those in the placebo group, the patients in the four-channel group showed significantly greater improvements in their average PASS (*p* ≤ 0.001), BBA (*p* ≤ 0.05), BBS (*p* ≤ 0.01), FMA (*p* ≤ 0.01), and MBI (*p* ≤ 0.001) results. Their average MBI results were also significantly better (*p* ≤ 0.01) than those of the dual-channel group. After the three weeks of treatment, the PASS, BBS, FMA, and MBI scores had all significantly improved further in the four-channel group (*p* ≤ 0.01, *p* ≤ 0.01, *p* ≤ 0.001, and *p* ≤ 0.001, respectively). By that point, the average MBI assessment in the dual-channel group was significantly better than that in the placebo group (*p* ≤ 0.01). Compared to the dual-channel group, the four-channel group did not perform significantly better on the FMA. These results are summarized in Table 2.

No significant FA difference between the groups was observed as ipsilateral or contralateral to the lesions at baseline. The ipsilateral FA showed significant increases after three weeks of intervention in all three groups. The four-channel and dual-channel groups both had significantly higher average FA values than at baseline after three weeks (*p* ≤ 0.05 in both cases); however, the placebo group showed no significant improvement. No significant differences were found in comparing the FA values among the three groups on the ipsilateral or contralateral side. The decreased rates of change on the ipsilateral and contralateral sides were also not significantly different, though the FA change on the ipsilateral side had changed significantly (*p* ≤ 0.05) comparing the four-channel group with the placebo group or the dual-channel group against the placebo group. The change in the four-channel and dual-channel groups was not significantly different. The rate of the change value in the ipsilateral FA also was not significantly different among the three groups except when the four-channel group was compared with the placebo group (*p* ≤ 0.05). These results are summarized in Table 3.

**Figure 2:**
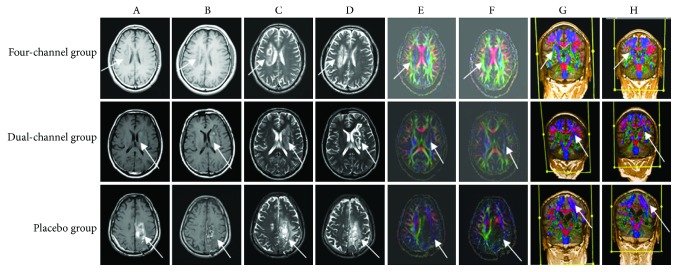
Representative brain remodeling with three subjects. (a), (c), (e), and (g) show T1WI, T2W2, FA, DTT, respectively, before intervention. (b), (d), (f), and (h) show T1WI, T2W2, FA, and DTT, respectively, after intervention. In all the three groups, pictures (b) and (d) suggest that perilesional edema was absorbed obviously after intervention; pictures (e) and (g) suggest that the bundle of fibers in lesional regions was significantly less than contralateral side and it was interrupted, compressed, and shifted. In the four-channel group, pictures (f) and (h) suggest that fibers in the same lesional regions increased significantly after treatment. In the dual-channel group, pictures (f) and (h) suggest that fibers in lesional regions do not increase but even decreased after treatment. Fibers seem to increase in the contralateral regions. In the placebo group, pictures (f) and (h) suggest that fibers in the lesional regions do not increase after treatment. We took the samples as follows: a patient, male, 62 years old, with left parietal lobe hematoma for the four-channel group; a patient, male, 52 years old, with right ganglia and corona radiata infarction for the dual-channel group; and a patient, male, 62 years old, with left parietal lobe hematoma for the placebo group.

Magnetic resonance images (Figure 2) showed lesions and perilesional edema in all three groups, but all were absorbed to some extent after the three weeks of treatment. DTI tractography indicated an increase in integrity after three weeks of four-channel treatment. There were more fibers in the ROI, and the diameter of the fiber bundles was enlarged in and around the lesions. New CST fibers projecting progressively closer to the motor cortex appeared during training. The dual-channel and placebo subjects had no obvious fiber changes.

## 4. Discussion

This study assessed brain remodeling induced by patterned FES in early-stage stroke patients. The motor function assessments showed motor recovery in response to all the interventions tested, but the main findings suggest that a four-channel FES mimic to gait can promote neuroplasticity which is helpful for motor recovery.

All of this study's subjects showed significant improvement in all of the outcome measures after 3 weeks of treatment. The between-group comparisons show that the four-channel group began to show a significant effect in terms of the MBI assessment at week 1 (compared to the placebo group) and began to achieve significantly better PASS and MBI results than the dual-channel and placebo groups by week 2. Average FMA scores significantly better than those in the dual-channel or placebo group appeared by week 3. The four-channel FES had early and significant effect on improving activities of daily living (ADL) from the assessment of MBI, which suggests that this FES based on a gait pattern could arise quality and dependency of patients' lives and also might help them return to community lives. Higher PASS score could illustrate a better sit-stand transferring ability and may indicate lower risk of fall or even bone fracture. PASS and FMA results showed better ability of posture control and even the whole lower extremities gained by four-channel FES. Although we always know that the “ceiling effect” and “floor effect” of the assessment scales may bring some limitations and errors, these results above still suggest that this programmed four-channel FES technique appears to be a more effective approach to improving posture control, balance, and walking ability of lower extremities of hemiplegic patients.

This study's protocol allowed the subjects to perform walking movements earlier, while they were still unable to sit, stand, or walk independently. This is a rational method for FES application in lower extremity rehabilitation. Some therapists emphasize efficiency and coordination in retraining stroke patients in adopting exercise mimic to complicated and integral movements rather than simple or local ones by instruments even in conditions of weight bearing [17]. In a power saving aspect, the power output from a limb is limited by the coordination pattern of the muscles rather than the maximum power output from any one of them [32]. In a sense input aspect, the entire lower extremity movements could bring more joints and muscles in functional movements to increase more proprioception input. Many researchers have tried to combine multiple-electrode FES with other gait training technologies (treadmill training, a gait robot, or motorized training) to make FES training more physiologically realistic [33, 34]. Treadmill training induces cycling movements; the kinematic pattern of which is very similar to that of walking, but cycling exercise requires less balance capacity. Both walking and cycling exercises require alternate activation of antagonists in the lower extremities in a well-coordinated manner. Repetitive cycling movement can increase range of motion and reduce the tone in hypertonic joints [35, 36]. Cycling feedback has been shown to promote neuromuscular control of the affected leg to establish normal patterns of gait [37, 38]. Four-channel FES allow the lower extremity to mimic gait pattern movements, bringing more sense input of locomotor, coordinating muscles, and upgrade the mechanical efficiency of limb movements. FES robot systems which involve a robot controlled by the subject's motor intention have brought in a new epoch in FES [33]; it could be more close to really functional activity. The simpler technique applied in this study—just using FES to induce gait-like action in a side-lying position—can be applied early after stroke even with a patient with poor cardiopulmonary function. Cyclic stimulation with the timing of a normal gait cycle aroused more coordinated movements of the muscles and joints. Repeated stimulation may constitute meaningful sensory and motor input to the brain, which may promote desirable brain plasticity.

The rationale for using fMRI in this study was that it provides anatomical as well as functional information. Functional MR depicts functional plasticity, but DTI has the advantage of addressing plasticity in the brain's structure directly by depicting changes in the number of fibers, their length, and their density [39]. DTI was used here to interpret the spatial heterogeneity and plasticity after the FES. As the results show, FA clearly decreased compared to the contralateral side before the FES treatments. This suggests that FA is sensitive enough to help clinical diagnosis and prediction of motor function in the early stage of stroke. FA on the ipsilateral side increased in all three groups after three weeks of intervention, but the magnitudes and rates of FA change suggest that four-channel FES was significantly more effective than dual-channel stimulation (or placebo treatment).

Diffusion tensor tractography (DTT) enables clear visualization of various fibers inside the white matter of the brain that are not visible with conventional imaging, making it a useful diagnostic tool. The integrity of the corticospinal tract also predicts motor function potential [25, 40, 41]. It has been shown by intraoperative fiber stimulation that the tracts seen using DTT reflect to some extent the functioning white matter fibers. But if the tracts do not appear on the image, that does not always mean that the fibers do not exist [42]. FA can also indicate the situation of fiber connections. As the DTT results show, after four-channel FES treatment, nerve fiber bundles increased significantly in number in the region ipsilateral to the lesion. The broken nerve fibers tended to reconnect. Dual-channel stimulation did not induce any obvious changes in the DTT results from the ipsilateral regions. Four-channel FES induced better-coordinated movement of the muscles and joints, and constraint-induced movements persisted longer, which could have led to the more obvious changes in brain plasticity shown through DTT. The brain regions around and in lesions are considered the main loci requiring functional fiber reconnection, but the contralateral side also showed plastic changes after treatment, though the changes were not considered significant in any of the three groups.

The matching of DTI and CST reconstruction was nearly consistent and reflected motor function recovery in this study. As illustrated, to innervate muscles of the more affected leg, the key may be not the quantity of CST fibers maintained but the intrinsic quality of these fibers. To investigate quality of the fibers, a DTI analysis and a delineation of CST fibers are useful. The interest of such information resides in the potential four-channel FES treatment that could be proposed as the function of the cortical organization and brain network reconnection. We will go on to find proper neuroimaging changes by fMRI to further explore the mechanisms of FES.

## 5. Conclusion

Four-channel and dual-channel FES applied soon after stroke can both improve motor function, balance, walking ability, and performance in the activities of daily living. Four-channel FES inducing walking-like leg motion may be more effective than dual-channel FES. It can improve motor function by promoting brain plasticity.

## Figures and Tables

**Table 1:** Subject demographics.

	FES-4	FES-2	FES-P
	*n* = 18	*n* = 15	*n* = 15
Age (years)	59 ± 11	60 ± 9	59 ± 9
Males	9 (50)	9 (60)	9 (60)
Females	9 (50)	6 (40)	6 (40)
Paretic side: right	11 (61.1)	6 (40)	7 (47)
Paretic side: left	7 (38.9)	9 (60)	8 (53)
Type of stroke: hemorrhagic	14 (77.8)	12 (80)	4 (26.7)
Type of stroke: ischemic	4 (22.2)	3 (20)	11 (73.3)
Time since stroke onset (days)	20 ± 11	21 ± 13	20 ± 12
Brunnstrom stage			
I	8 (44.4)	6 (40)	5 (33)
II	7 (38.9)	7 (46.7)	8 (53.3)
IV	3 (16.7)	2 (13.3)	2 (13.3)

Values are mean ± SD or number (percent of the group).

**Table 2:** Comparison of assessment outcomes among the 3 groups.

Variable	Case number	Group	Week 0	Week 1	Week 2	Week 3	Change after 1 week	Change after 2 weeks	Change after 3 weeks
PASS	18	Four-channel	11 ± 5	23 ± 6^a^	29 ± 3^a^	31 ± 3^a^	12 ± 5	18 ± 4	20 ± 5
	15	Dual-channel	12 ± 8	20 ± 9^a^	24 ± 8^ab^	28 ± 8^a^	8 ± 5^b^	12 ± 5^b^	16 ± 4^b^
	15	Placebo	12 ± 8	20 ± 10^a^	21 ± 7^ab^	25 ± 8^ab^	8 ± 4^b^	8 ± 5^bc^	13 ± 5^b^
BBA	18	Four-channel	3.1 ± 1.3	5.0 ± 1.9^a^	8.3 ± 2.4^a^	9.2 ± 2.3^a^	2.0 ± 1.5	5.2 ± 2.6	6.1 ± 2.5
	15	Dual-channel	3.5 ± 1.6	4.8 ± 2.6^a^	6.9 ± 3.0^a^	8.7 ± 3.0^a^	1.3 ± 1.5	3.4 ± 1.8^b^	4.7 ± 1.7^b^
	15	Placebo	3.1 ± 2.2	5.1 ± 3.3^a^	6.2 ± 3.1^ab^	8.0 ± 3.5^a^	1.9 ± 1.6	3.1 ± 1.2^b^	4.9 ± 1.8
BBS	18	Four-channel	6 ± 5	20 ± 11^a^	37 ± 7^a^	43 ± 8^a^	14 ± 9	31 ± 7	37 ± 8
	15	Dual-channel	6 ± 5	18 ± 15^a^	29 ± 16^a^	37 ± 13^a^	12 ± 12	23 ± 12^b^	31 ± 10^b^
	15	Placebo	9 ± 10	18 ± 15^a^	24 ± 16^ab^	29 ± 17^ab^	10 ± 8	16 ± 9^bc^	21 ± 11^bc^
FMA	18	Four-channel	9 ± 6	15 ± 4^a^	22 ± 5^a^	25 ± 5^a^	6 ± 5	13 ± 7	16 ± 8
	15	Dual-channel	8 ± 4	13 ± 7^a^	18 ± 9^a^	20 ± 7^a^	5 ± 4	9 ± 5	12 ± 5
	15	Placebo	8 ± 6	13 ± 8^a^	15 ± 8^ab^	17 ± 9^ab^	5 ± 5	7 ± 7^b^	9 ± 6^b^
MBI	18	Four-channel	22 ± 9	52 ± 12^a^	73 ± 13^a^	81 ± 13^a^	30 ± 9	51 ± 11	59 ± 11
	15	Dual-channel	23 ± 13	44 ± 20^a^	60 ± 17^ab^	71 ± 15^a^	21 ± 10^b^	37 ± 12^b^	48 ± 7^b^
	15	Placebo	24 ± 13	38 ± 18^abc^	47 ± 20^ab^	54 ± 25^abc^	15 ± 9^b^	23 ± 10^bc^	31 ± 15^bc^

Values are mean ± SD. Change is the value before treatment minus the one at the time indicated. ^a^Significant difference at the *p* ≤ 0.001 level of confidence compared with the baseline value. ^b^Significant difference at the *p* ≤ 0.05 level of confidence compared with the four-channel group. ^c^Significant difference at the *p* ≤ 0.05 level of confidence compared with the dual-channel group.

**Table 3:** Comparison of FA outcomes among the 3 groups.

Group	Case number	Ipsilateral FA value		Contralateral FA value		Change value		Rate of change value	
		Week 0	Week 3	Week 0	Week 3	Ipsilateral FA change	Contralateral FA change	Ipsilateral rate	Contralateral rate
Four-channel	6	0.155 ± 0.087	0.321 ± 0.172^a^	0.585 ± 0.058	0.588 ± 0.068	0.166 ± 0.111	0.002 ± 0.038	0.073 ± 0.017	0.044 ± 0.034^a^
Dual-channel	6	0.186 ± 0.076	0.333 ± 0.164^a^	0.600 ± 0.1	0.590 ± 0.124	0.147 ± 0.091	−(0.009 ± 0.106)	0.069 ± 0.011	0.043 ± 0.021^a^
Placebo	6	0.185 ± 0.12	0.217 ± 0.135	0.558 ± 0.123	0.562 ± 0.129	0.032 ± 0.052^bc^	0.065 ± 0.118	0.061 ± 0.023	0.060 ± 0.025^bc^

Value: mean ± SD; change value: FA values of week 3 minus those of week 0; rate of change value: (ipsilateral FA − contralateral FA)/ipsilateral FA^∗^100%. ^a^*p* < 0.05 indicates significant difference when compared with week 0 within groups. ^b^*p* < 0.05 indicates significant difference when compared with the four-channel group. ^c^*p* < 0.05 indicates significant difference when compared with the dual-channel group.

## Data Availability

The data used to support the findings of this study are available from the corresponding author upon request.
